# Reversible Room Temperature H_2_ Gas Sensing Based on Self-Assembled Cobalt Oxysulfide

**DOI:** 10.3390/s22010303

**Published:** 2021-12-31

**Authors:** Hui Zhou, Kai Xu, Nam Ha, Yinfen Cheng, Rui Ou, Qijie Ma, Yihong Hu, Vien Trinh, Guanghui Ren, Zhong Li, Jian Zhen Ou

**Affiliations:** 1 Key Laboratory of Advanced Technologies of Materials, Ministry of Education, School of Materials Science and Engineering, Southwest Jiaotong University, Chengdu 610031, China; zlfhb_zh@163.com (H.Z.); chengmo.19861208@163.com (Y.C.); zhong.li@swjtu.edu.cn (Z.L.); jianzhen.ou@rmit.edu.au (J.Z.O.); 2 School of Engineering, RMIT University, Melbourne, VIC 3000, Australia; nam.ha@rmit.edu.au (N.H.); s3465168@student.rmit.edu.au (R.O.); s3641260@student.rmit.edu.au (Q.M.); s3647679@student.rmit.edu.au (Y.H.); s3618890@rmit.edu.vn (V.T.); guanghui.ren@rmit.edu.au (G.R.)

**Keywords:** cobalt oxysulfide, H_2_ sensor, physisorption, room-temperature gas sensing

## Abstract

Reversible H_2_ gas sensing at room temperature has been highly desirable given the booming of the Internet of Things (IoT), zero-emission vehicles, and fuel cell technologies. Conventional metal oxide-based semiconducting gas sensors have been considered as suitable candidates given their low-cost, high sensitivity, and long stability. However, the dominant sensing mechanism is based on the chemisorption of gas molecules which requires elevated temperatures to activate the catalytic reaction of target gas molecules with chemisorbed O, leaving the drawbacks of high-power consumption and poor selectivity. In this work, we introduce an alternative candidate of cobalt oxysulfide derived from the calcination of self-assembled cobalt sulfide micro-cages. It is found that the majority of S atoms are replaced by O in cobalt oxysulfide, transforming the crystal structure to tetragonal coordination and slightly expanding the optical bandgap energy. The H_2_ gas sensing performances of cobalt oxysulfide are fully reversible at room temperature, demonstrating peculiar p-type gas responses with a magnitude of 15% for 1% H_2_ and a high degree of selectivity over CH_4_, NO_2_, and CO_2_. Such excellent performances are possibly ascribed to the physisorption dominating the gas–matter interaction. This work demonstrates the great potentials of transition metal oxysulfide compounds for room-temperature fully reversible gas sensing.

## 1. Introduction

Gas sensors have been an effective tool in monitoring gaseous pollutants, industrial production and household safety, greenhouse gas emission, and human health [1,2,3]. The emerging Internet of Things (IoT) technologies, in which the sensor devices serve as the foundation layer, require the new generation gas sensors with advantageous features of low-power consumption and low cost, while their sensitivity and selectivity should be maintained and improved in reference to traditional counterparts [4,5]. Solid-state semiconducting devices, relying on the surface interaction of core materials with gas molecules, have been one of the most popular gas sensors due to their high sensitivity, low cost, and long-term stability, paving a promising pathway to be adapted with the IoT technologies [6]. Metal oxides (e.g., SnO [7,8,9,10,11,12], ZnO [13,14,15,16,17,18], WO_3_ [19,20,21,22,23,24], TiO_2_ [12,14,25,26] etc.) have been the most studied category of core-sensitive materials in semiconducting gas sensors. However, their gas sensing performances are normally observed at elevated temperatures which allow for sufficient energy for the interaction of surface-adsorbed O with the target gas molecules [7,10,11,15,16,18,19,21,22,26]. For the case of the room temperature operation condition, the recovery phase of the many intrinsic metal oxide-based sensors, particularly with two-dimensional or high-dimensional structures, was either incomplete or with extremely slow kinetics [17,24,27]. Furthermore, the elevated temperature-driven chemisorption exhibits relatively poor selectivity of target gas molecules, significantly drawing back the overall gas sensing performances [12,25]. So far, there have been limited reports on room-temperature fully reversible sensing of oxidating gases (particularly NO and NO_2_) [24,26], while reducing gases (e.g., H_2_) are difficult to achieve without the incorporation of metallic catalysts. Therefore, the exploration of alternative semiconducting candidates has been an ongoing request.

Metal sulfides, with the two-dimensional (2D) or ultra-thin morphology, have recently attracted great attention for developing selective and reversible gas sensors at much lower operating temperatures [7,27,28,29,30,31,32]. Within this group of materials, the physical adsorption (or called “physisorption) of gas molecules dominates the surface interaction rather than the conventional chemisorption mechanism, in which electrical dipoles are formed on the surface of the host material as a result of interfacial charge transfer directly with the physisorbed gas molecules [3,27]. The response strength correlates with the adsorption energy of the material towards gas molecules as well as the relative band positions of the host material with the molecular orbitals of the target gas [3,31,33,34]. Without the involvement of catalytic reactions of chemisorbed O, physisorption requires minimum energy to activate and exhibit a relatively high degree of selectivity towards the target gas [3,33,35,36]. So far, reversible gas sensors based on ultra-thin metal sulfides such as MoS_2_ [36], WS_2_ [37], SnS_2_ [3,27], and SnS [38] have been reported at low elevated temperatures (<150 °C) or room temperature under light excitation. Nevertheless, the intrinsic room temperature fully reversible room-temperature gas sensing response has not been achieved without the external stimulus, including light excitation and voltage biasing. In addition, the surface of metal sulfides is sensitive to oxygen and oxidation may occur in the long run of gas sensing tests, causing unexpected drifts and performance variations [38,39,40].

Metal oxysulfides are intermediates during the transition from metal sulfides to oxides, which have recently received attention for their strong potentials in gas sensing [41]. Within the metal-sulfide framework, part of the sulfide atoms is replaced by oxygen atoms through power-intensive approaches (e.g., calcination and probe-sonication) [41,42], resulting in the modification of the electronic band structure and more importantly the improvement of long term stability [41,42]. Furthermore, physisorption remains the dominant gas adsorption interaction with the oxysulfides. For example, ultra-thin Janus indium oxysulfide (InS_x_O_y_)/indium sulfide (In_2_S_3_), synthesized from the power-intensive liquid-phase exfoliation of bulk In_2_S_3_ bulk crystals, exhibits a fully reversible, room temperature, and highly selective NO_2_ gas sensing performances under the visible light excitation, with an extremely low limit of detection of 0.363 ppb [39]. Recently, 2D palladium oxysulfide (PdSO_4_), as a representative within the category of transition metals, is realized from the probe-sonication of palladium sulfide (PdS) crystals in the liquid form [40]. The corresponding reversible NO_2_ sensing response occurs at room temperature without the implementation of light excitation.

In this work, we extend the exploration of the intrinsic room temperature sensing performances of ultra-thin metal oxysulfide towards reducing gases such as H_2_. Room-temperature fully reversible H_2_ gas sensing is highly desirable given the rapid development of zero-emission vehicles and fuel cells. Here, we select self-assemble cobalt sulfide (CoS) made of ultra-thin nanoflakes as the initial materials. Upon the calcination at an elevated temperature, the transformation of CoS into cobalt oxysulfide (CoS_x_O_y_) occurs, in which the hierarchical morphology, crystal structure, chemical composition, and band structure are revealed. The room temperature gas sensing performances of cobalt oxysulfide are investigated in air-balanced H_2_ gas and a comparative study is carried out against other commonly seen oxidating and reducing gases including CH_4_, NO_2_, and CO_2_. Finally, the related gas sensing mechanism is discussed.

## 2. Materials and Methods

### 2.1. Material Synthesis and Preparation

The cobalt sulfide was synthesized by mixing 286 mg of Cobalt (II) chloride hexahydrate (Cl_2_CoH_12_O_6_) (>99.0%) (Chron Chemicals Co. Ltd., Chengdu, China) and 274 mg of Thiourea (CH_4_N_2_S) (>99.0%) (Chron Chemicals Co. Ltd., Chengdu, China) in 30 mL of deionized water. After a vigorous string (500 revolutions per minute (rpm)) at 30 °C for 30 min, the mixture was autoclaving at 180 °C for 18 h. Followed by cooling down to room temperature, the obtained solution was centrifuged (5000 rpm) for 20 min to collect the precipitate for another 20 min of centrifugal washing (5000 rpm) in deionized water. Finally, the cobalt sulfide powder was collected by drying up the collected precipitate at 50 °C for 24 h; the cobalt oxysulfide was prepared by annealing the synthesized cobalt sulfide powder at 600 °C (ramping up by 300 °C per hour) with a constant flowrate (197 standard cubic centimeter per minute (sccm)) of compressed dry air under one standard atmosphere. After cooling down (ramping down by 300 °C per hour), the cobalt oxysulfide was obtained and dispersed in 10 mL of ethanol.

### 2.2. Material Characerizations

An FEI Nova NanoSEM 200 was used to investigate the micro-cage structure of cobalt oxysulfide. The crystal lattices and chemical composition of the material were studied under a JEOL JEM-F200 transmission electron microscopy (TEM) with an energy-dispersive X-ray spectroscopy (EDS) detector equipped (accelerating voltage of 200 kV). X-ray diffraction (XRD) measurements were conducted on a Bruker D4 ENDEAVOR with a monochromatic Cu Kα radiation source (λ = 0.154 nm) equipped. X-ray photoelectron spectroscopy (XPS) was performed on a Krato AXIS Supra XPS (dual Al/Ag monochromatic X-ray source equipped) using Al Kα X-rays at 1486.7 eV. The measured XPS spectra were analyzed using CasaXPS (version 2.3.24). The material optical absorption property was investigated using a Cary 500 spectrometer, in which the UV-Vis-NIR spectra were measured on a drop-casted material sample upon a glass substrate. A HORIBA LabRAM HR Evolution was utilized to study the Raman spectra of cobalt oxysulfide with the excitation wavelength of 532 nm.

### 2.3. Sensor Fabrication and Measurements

Upon an interdigital transducer (IDT) substrate with 200 pairs of gold electrodes (HORX Sensortech, Rowville, VIC, Australia), 10 µL of cobalt oxysulfide solution was drop-casted, forming a gas sensing unit. A customized gas chamber was utilized for the gas sensing experiments, in which the resistance of the sensor was kept monitoring on an Agilent 34401A digital multimeter (Keysight Technologies, Mulgrave, VIC, Australia). Meanwhile, a computerized multichannel gas calibration system was applied to regulate an income gas stream to the gas chamber with a constant flowrate (~100 sccm).

## 3. Results and Discussion

**Figure 1:**
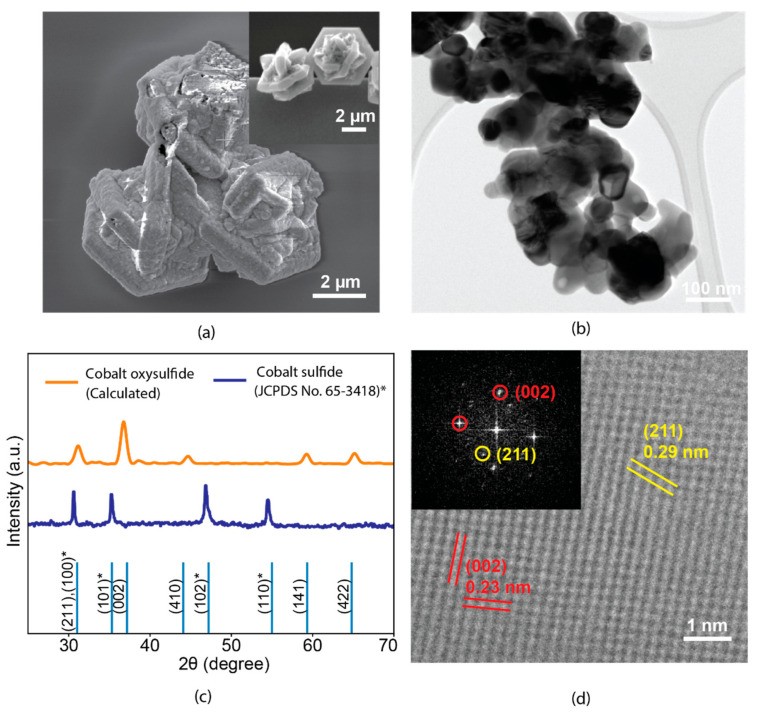
Morphology and crystal lattice structure for cobalt oxysulfide in (**a**) SEM and (**b**) low resolution TEM, (**c**) XRD (planes marked with black star ‘*’ belong to cobalt sulfide (JCPDS No. 65-3418)), and (**d**) HRTEM.

According to the scanning electron microscope (SEM) images shown in Figure 1a and the inset, initial CoS exhibited a micro-cage morphology self-assembled by the hexagonal sheets. The elevated temperature annealing of cobalt sulfide maintained its micro-cage morphology compared to that of the initial sample. However, the hexagonal shape was distorted and granular structures appeared on the surface of the cage, suggesting the transformation of the crystal structure. Such a hierarchical microstructure was self-assembled from spherical flakes with sizes of ~100 nm, as demonstrated by the low-resolution TEM in Figure 1b. A significant portion of the nanoflakes exhibited a high degree of transparency, indicating their relatively small thicknesses. The crystal transformation was confirmed by the XRD pattern. From Figure 1c, the feature peaks of hexagonal cobalt sulfide at 31.3°, 35.3°, 47.2° and 55.0° can be ascribed as the crystal plane of (100), (101), (102), and (110), which is in good agreement with the reported literatures [41,42]. Such a crystal structure was transformed to an orthorhombic system (space group *Pnma*, a = 8.62400 Å, b = 6.71500 Å, and c = 4.74400 Å) after the annealing treatment, showing the distinct peaks at 31.3°, 36.8°, 44.8°, 59.3°, and 65.2°, matching well with the simulated lattice plane of (211), (002), (410), (141), and (422) of cobalt oxysulfide [43]. Furthermore, a high-resolution TEM (HRTEM) image in Figure 1d reveals the atomic structure of the material. Two clear sets of lattice spacings, which were 0.23 and 0.29 nm, reflect the (002) and (211) crystal planes, respectively. Such an observation can also be found in the corresponding fast Fourier transform (FFT) pattern (inset of Figure 1d), indicating the crystal transformation from an initially hexagonal into tetragonal transformation.

**Figure 2:**
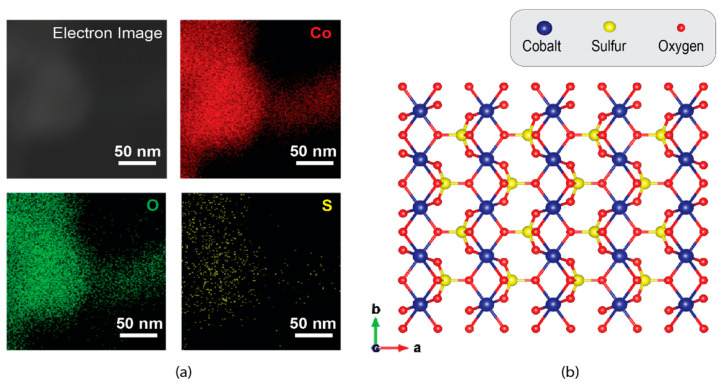
(**a**) Elemental analysis of cobalt oxysulfide on EDS. (**b**) The theoretical crystal model of cobalt oxysulfide (based on VESTA 3.5.7).

**Figure 3:**
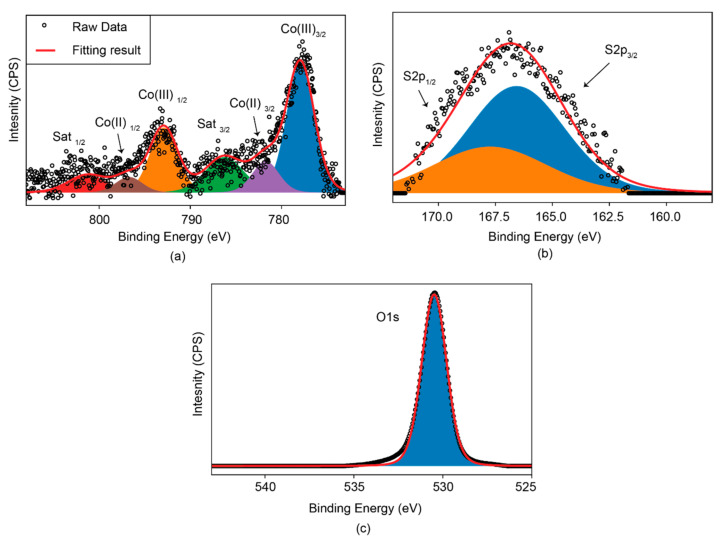
XPS analysis of cobalt oxysulfide for (**a**) Co 2p, (**b**) S 2p, and (**c**) O 1s.

The localized chemical composition of the cobalt oxysulfide sample was assessed using the EDS detector integrated within the TEM. As shown in Figure 2a, elements of Co, O, and S were identified within an individual nanoflake which was used for the self-assembly of the micro-cage structure. It is noted that the content of S was significantly smaller than that of O, suggesting that the majority of S sites were replaced by O during the calcination. Such a phenomenon is not observed in that of hexagonal cobalt sulfide (Figure S1). This observation indeed consists of the theoretical modeling of cobalt oxysulfide [43] (Figure 2b), in which only one sulfide atom was bonded with four oxygen atoms. In addition, the elemental analysis was extended to the overall sample area using XPS. From Figure 3a, there are six deconvoluted peaks observed in the Co 2p spectrum. The predominant peaks located at 777.9 and 792.9 eV are ascribed to the Co^3/2^ and Co^1/2^ of the 3+ oxidation states, respectively [44]. In addition, the peaks at 781.7 and 796.7 eV both indicate the Co^2+^ oxidation states [44,45,46], while the rest at 786.3 and 801.3 eV are typical satellite features within the Co spectrum [44,45,46]. The S 2p spectrum of cobalt oxysulfide is shown in Figure 3b, wherein the conventional S 2p peaks in the region between 161 and 163 eV are missing. Instead, a broad peak centered at ~167 eV together with a shoulder at ~168 eV represent the S^3/2^ and S^1/2^ of the S-O bond, respectively [41,46], providing another direct evidence of the formation of the oxysulfide compound. Figure 3c indicates the O 1s spectrum of cobalt oxysulfide, in which there is one sharp peak centered at 531 eV that is originated from the Co-O bond mixed with the silica substrate signal [44].

**Figure 4:**
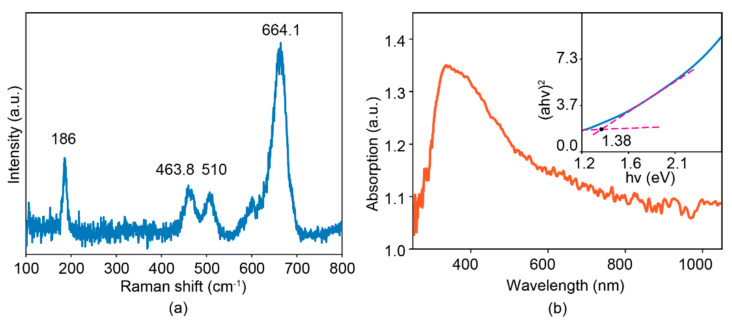
Raman spectrum (**a**). UV-Vis-NIR absorption spectra (**b**) with the corresponding Tauc-plot in the inset.

Raman spectroscopic measurement was further taken to investigate the bonding information of cobalt oxysulfide. From Figure 4a, an intensified peak at 186 cm^−1^ is observed, which can be assigned to the symmetrical vibration mode of Co-O tetrahedrons [44]. Another peak at 664.1 cm^−1^ exhibited a 12 wavenumber blue-shift compared to that of pure hexagonal CoS [47], possibly ascribed to the bond vibration of Co-SO. Similar blue shifts were seen at the doublet located at 463.8 and 510 cm^−1^, which are 11 and 7 wavenumbers shifted compared to those of hexagonal CoS and tetragonal Co_3_O_4_, respectively [44,47]. We consider that such a doublet also represents the feature of the Co-SO bonding vibration. The optical absorption measurements of the sample were carried out to estimate its optical bandgap. From Figure 4b, the main absorption peak is found to be ~380 nm with an extended edge across the whole visible light spectrum. The Tauc plot, derived from the value of (αhv)^2^ against the optical energy hv [3,43,48,49], is shown in the inset of Figure 4b, revealing an optical bandgap energy of ~1.38 eV. Such a value is around 0.13 eV larger than that of pure hexagonal CoS (Figure S2) possibly due to the replacement of S with O. A similar observation was found in the case of indium oxysulfide derived from indium sulfide [41].

**Figure 5:**
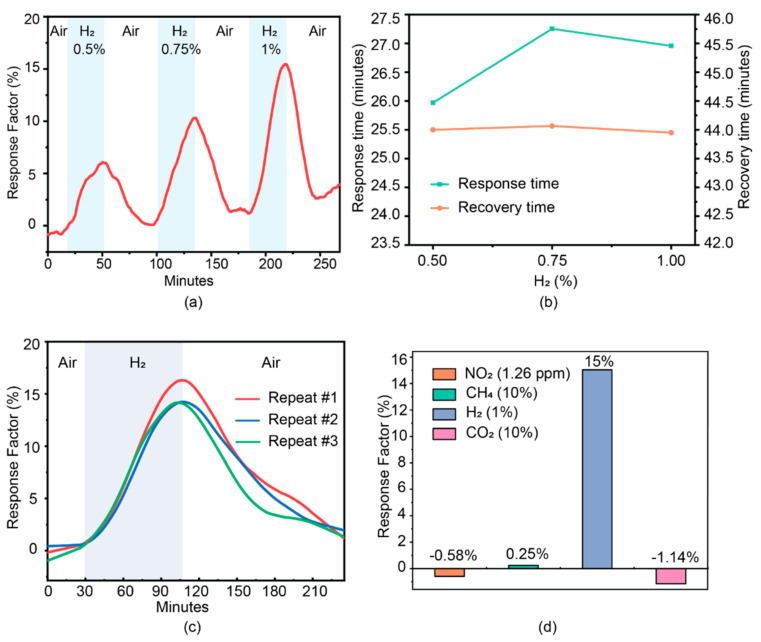
Gas sensing behaviors of the cobalt oxysulfide sensor at room temperature with (**a**) a dynamic response, (**b**) response/recovery time along with H_2_ concentration, (**c**) repeatability experiments at 1% of H_2_, and (**d**) a selectivity comparison between NO_2_ (1.26 ppm), CH_4_ (10%), H_2_ (1%), and CO_2_ (10%).

To assess the gas sensing performance of the self-assembled cobalt oxysulfide micro-cages, a 10 µL of suspension containing 5 µg of material was drop-casted into the transducing substrate containing 200 pairs of interdigital electrodes (IDE) with the spacing of 10 μm. The sensor was placed in a customized gas testing chamber and its electrical resistance was measured by a desktop multimeter through a minimized probe stage within the chamber. The response factor was determined by the formula of (R_target_ − R_air_)/R_air_ × 100%, in which R_target_ is the resistance upon the exposure of the target gas and R_air_ is the resistance in the air. In addition, the response and recovery time were obtained by the time difference between 90% of the full response magnitude and 10% of the full response magnitude. We firstly investigated the H_2_ gas sensing performances of the sensor at room temperature. By changing the mixing ratio between target gases and the compressed dry air at the gas chamber inlet, the concentration of hydrogen can be well controlled using programmable mass flow controllers. From Figure 5a, for the H_2_ gas concentration of 0.5%, the response factor reaches ~5% with a response time of ~26 min (Figure 5b). Such a room temperature response is fully reversible with a recovery time of ~44 min. Given the donor nature of H_2_ gas [50,51], the positive response factor reflects a typical p-type sensor response, implying the p-type semiconducting property of cobalt oxysulfide. Upon the increase of the H_2_ exposure concentration from 0.5% to 0.75% and 1%, the corresponding response factor was enhanced to 10% and 15% in an almost linear trend, respectively. However, the response time was slightly prolonged to ~27 min and saturated beyond the concentration of 0.75%, while the recovery time remained unchanged. The fully reversible H_2_ room temperature response also exhibited high repeatability, in which the response factor remained at ~10% for three consecutive runs at the H_2_ concentration of 1% (Figure 5c). In addition, we extended the investigation of gas sensing performances towards other commonly seen gases such as NO_2_ (1.26 ppm), CH_4_ (10%), and CO_2_ (10%), in which response factors of −0.58%, 0.25%, and 1.14% were obtained, respectively. Therefore, the H_2_ response factor is at least one order larger than those of the three gases, revealing a high degree of selectivity. From Figure S3, the cobalt oxysulfide sensor demonstrates an excellent long-term stability towards 1% H_2_ with negligible performance degradation during a week. Furthermore, the material was confirmed with consistent morphologies and crystal structures after the long-term test (Figure S4). The sensor was also tested towards 1% H_2_ at 40% relative humidity (RH). As shown in Figure S5, the response factor of cobalt sulfide sensor slightly drops ~4% in a humidified environment compared to that of the dry condition possibly due to the adsorption of water vapor [42].

Unlike the spill-over effect on which most catalyst-enabled H_2_ room temperature sensors are based [21,52], cobalt oxysulfide exhibits such a rarely seen fully reversible H_2_ sensing at room temperature without the implementation of catalysts and external stimulus (e.g., light, heat and voltage biasing). Therefore, we consider that the impressive response is originated from the intrinsic interaction of the adsorbed H_2_ gas molecules with the material. For the conventional chemisorption mechanism, the gas sensing performance is enabled by the catalytic interaction of chemisorbed O with the target gas molecules, producing an interfacial charge transfer which alters the electrical resistance of the material [3,29,43]. However, the chemisorption-driven room temperature reversible sensing has only been realized in oxidating gases such as NO_2_ [9,53,54,55]. In addition, crystal defects in nanostructures act as deep trap sites on reducing gases such as H_2_ at room temperature, resulting in the absence of a significant prolonging of the recovery phase [53,56]. Given the greatly enhanced response factor of H_2_ over NO_2_ in cobalt oxysulfide, we therefore believe that the gas sensing mechanism is dominated by physisorption rather than chemisorption. It has been reported that physisorption governs the gas interaction with 2D or ultra-thin materials due to their predominant surface properties over the bulk properties [3,29,34]. Physisorption relies on the direct interaction of adsorbed gas molecules with the material, without the involvement of chemisorbed O, in which the interaction depends on the surface adsorption energy for the target gas molecules as well as the relative band positions of the materials with the gas molecular orbitals for enabling the interfacial charge transfer [3,29,43]. In our case, H_2_ gas molecules were physisorbed on the surface of cobalt oxysulfide. Given the energy gap between the Fermi level of cobalt oxysulfide and the highest occupied molecular orbital (HOMO) level of the H_2_ gas molecule, electron density drifts from the adsorbed H_2_ to the material surface, subsequently leading to the Fermi-level pinning towards the molecular HOMO upon adsorption [54]. Such interfacial charge transfer results in electrical dipoles which are formed in the matter–gas interface, in which holes are attracted from the material body to the surface given the donor nature of H_2_ gas molecules. The charge redistribution therefore occurs in cobalt oxysulfide, decreasing the available number of holes within the material body and increasing the electrical resistance.

## 4. Conclusions

We successfully obtained nanostructured cobalt oxysulfide from the calcination of ultra-thin cobalt sulfide nanoflakes self-assembled into the hexagonal micro-cage morphology. Upon the annealing treatment, the micro-cage structure was maintained, while the hexagonal shape was distorted. Through TEM analysis, the cobalt oxysulfide micro-cages were composed of ultra-thin spherical nanoflakes with lateral dimensions of ~100 nm. Both the TEM-EDS and XPS results revealed that the majority of S atoms within the Co-S framework were replaced by O atoms, causing the crystal transformation from initially hexagonal to tetragonal coordination. In addition, the bandgap energy was slightly expanded to ~1.38 eV. As a result, the cobalt oxysulfide was tested with the room temperature H_2_ gas sensing performance without the implementation of light excitation and voltage biasing. A response magnitude of ~15% was found for 1% H_2_ gas balanced in the air with a high degree of repeatability and full reversibility. Furthermore, the response magnitude of 1% H_2_ was at least one order larger than those of commonly seen gases including NO_2_ (1.26 ppm), CH_4_ (10%), and CO_2_ (10%), demonstrating high selectivity towards H_2_, which was rarely seen in semiconducting gas sensors without the incorporation of metallic catalysts. Such fully reversible, highly selective, and room-temperature H_2_ gas sensing performance could be ascribed to the physisorption that governed the matter–gas interaction mechanism, which has been observed in many 2D metal sulfides. We consider that this work demonstrates further evidence regarding the high-performance room-temperature gas sensing properties of transition metal oxysulfide, serving as suitable candidates in developing next-generation gas sensors adaptable with IoT technology.

## Supplementary Materials

The following are available online at https://www.mdpi.com/article/10.3390/s22010303/s1, Figure S1: EDS measurement of the hexagonal cobalt sulfide particles upon a SiO2 substrate, Figure S2: UV-Vis-NIR absorption spectra of CoS with the corresponding Tauc-plot in the inset, Figure S3: Long term stability experiment for cobalt oxysulfide sensor towards 1% H2 for a week, Figure S4: (a) Low resolution TEM and (b) HRTEM images for cobalt oxysulfide taken after long-term stability test, Figure S5: The response curve of cobalt oxysulfide sensor towards 1% H2 in the dry, and 40% RH.
