# Supplementary material for: Reversible Room Temperature H2 Gas Sensing Based on Self-Assembled Cobalt Oxysulfide
Source: Sensors (Basel). 2021 Dec 31;22(1):303. doi: 10.3390/s22010303 (PMC8749549; doi:10.3390/s22010303)
Supplement: Supplementary file 1 [file sensors-22-00303-s001.zip › sensors-1522545-supplementary.pdf]

Supporting Information

Reversible room temperature H_2 gas sensing based on self-assembled cobalt oxysulfide

Hui Zhou [1], Kai Xu [2,*], Nam Ha [2], Yinfen Cheng [1], Rui Ou [2], Qijie Ma [2], Yihong Hu [2], Vien Trinh [2], Guanghui Ren [2], Zhong Li [1,*], Jian Zhen Ou [1,2,*]

[1] Key Laboratory of Advanced Technologies of Materials, Ministry of Education, School of Materials Science and Engineering, Southwest Jiaotong University, Chengdu 610031, China

[2] School of Engineering, RMIT University, Melbourne, Victoria 3000, Australia

[*] Correspondence: kai.xu@rmit.edu.au (K.X.); zhong.li@swjtu.edu.cn (Z.L.); jianzhen.ou@rmit.edu.au (J.Z.O.)

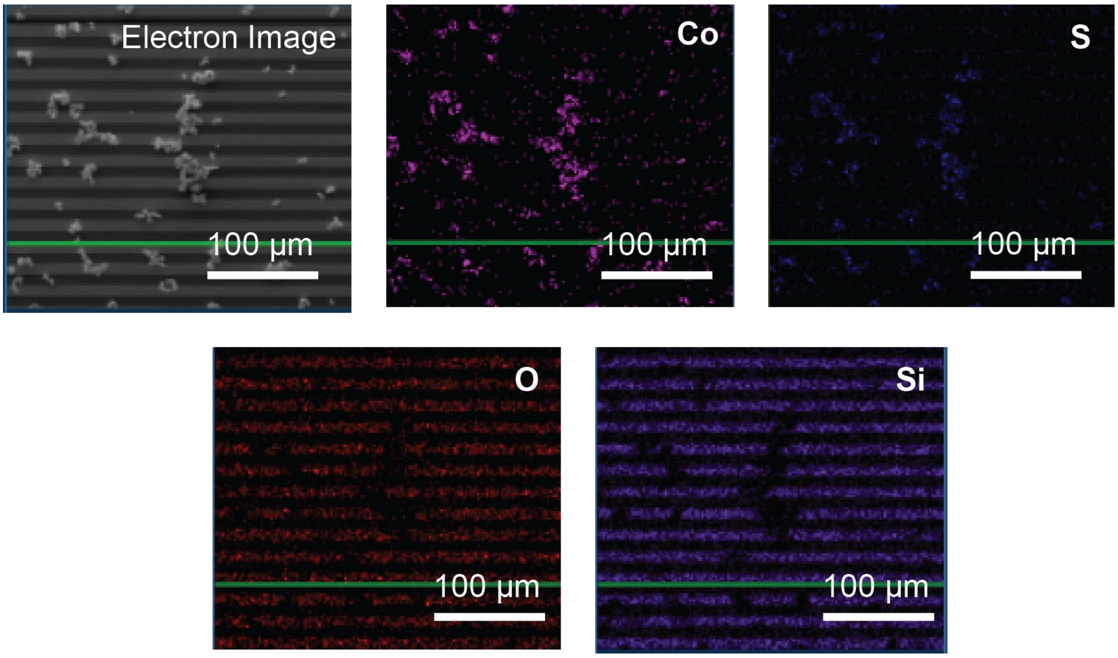

Figure S1. EDS measurement of the hexagonal cobalt sulfide particles upon a SiO_2 substrate.

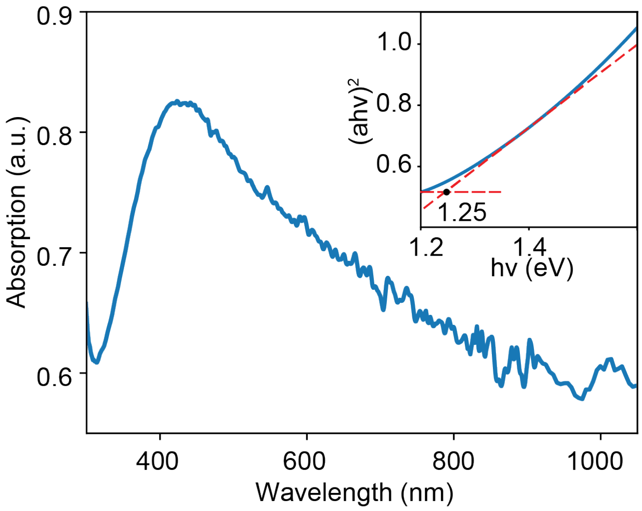

Figure S2. UV-Vis-NIR absorption spectra of CoS with the corresponding Tauc-plot in the inset.

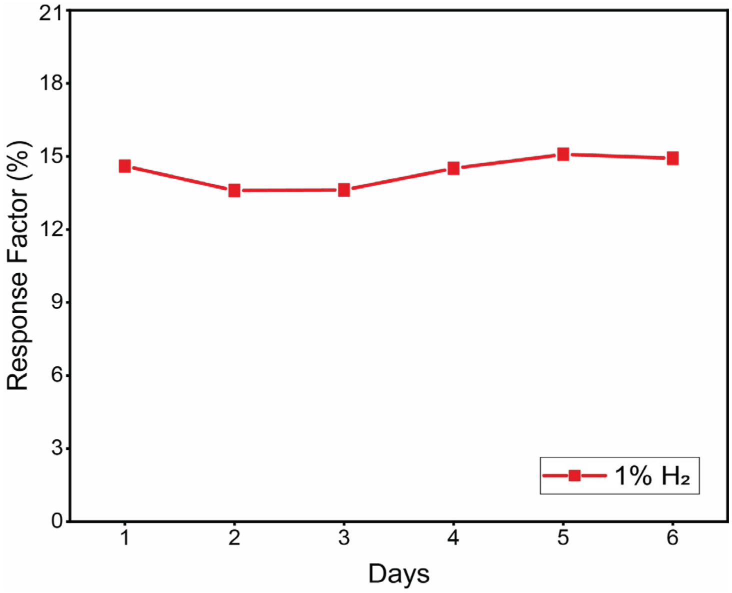

Figure S3. Long term stability experiment for cobalt oxysulfide sensor towards 1% H_2 for a week.

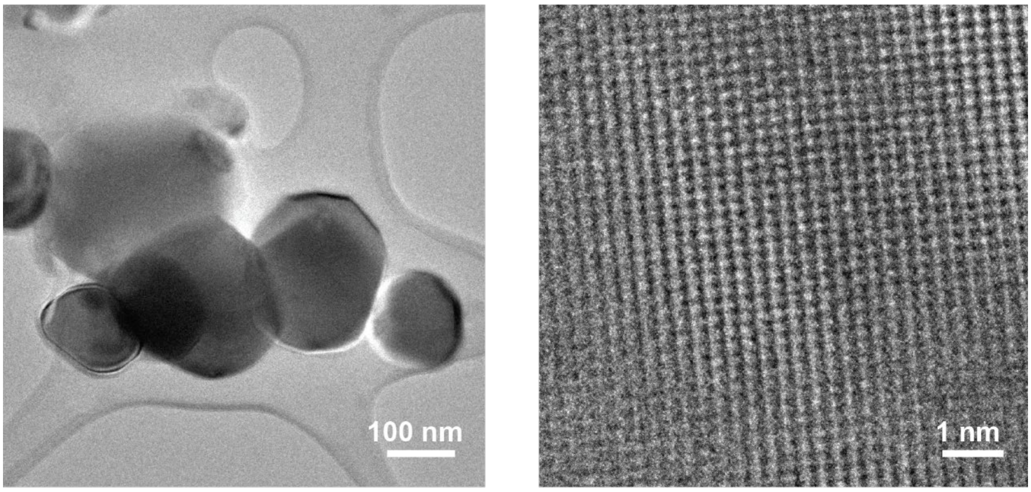

(a) (b)

Figure S4. **(a)** Low resolution TEM and **(b)** HRTEM images for cobalt oxysulfide taken after long-term stability test.

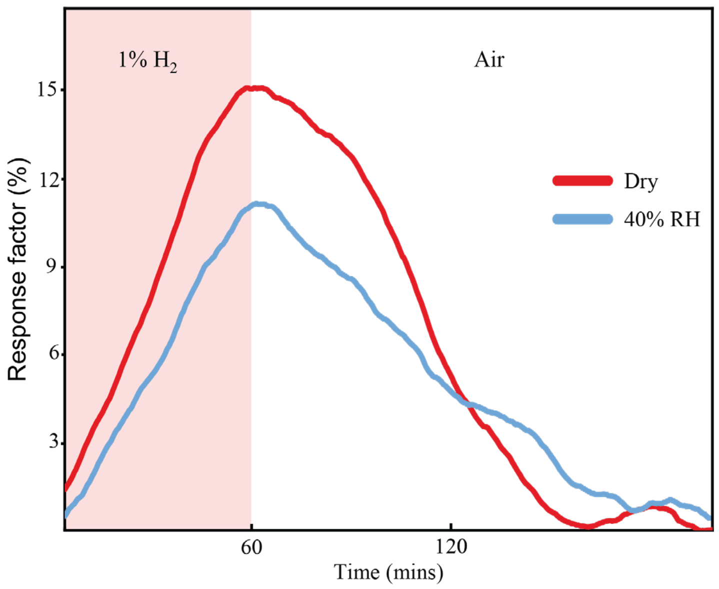

Figure S5. The response curve of cobalt oxysulfide sensor towards 1% H_2 in the dry, and 40% RH.
